# Regulation of System *x*_*c*_^−^ by Pharmacological Manipulation of Cellular Thiols

**DOI:** 10.1155/2015/269371

**Published:** 2015-04-09

**Authors:** Rebecca Albano, Nicholas J. Raddatz, Julie Hjelmhaug, David A. Baker, Doug Lobner

**Affiliations:** Department of Biomedical Sciences, Marquette University, 561 N. 15th Street, Room 426, Milwaukee, WI 53233, USA

## Abstract

The cystine/glutamate exchanger (system x_c_^−^) mediates the transport of cystine into the cell in exchange for glutamate. By releasing glutamate, system x_c_^−^ can potentially cause excitotoxicity. However, through providing cystine to the cell, it regulates the levels of cellular glutathione (GSH), the main endogenous intracellular antioxidant, and may protect cells against oxidative stress. We tested two different compounds that deplete primary cortical cultures containing both neurons and astrocytes of intracellular GSH, *L*-buthionine-sulfoximine (L-BSO), and diethyl maleate (DEM). Both compounds caused significant concentration and time dependent decreases in intracellular GSH levels. However; DEM caused an increase in radiolabeled cystine uptake through system x_c_^−^, while unexpectedly BSO caused a decrease in uptake. The compounds caused similar low levels of neurotoxicity, while only BSO caused an increase in oxidative stress. The mechanism of GSH depletion by these two compounds is different, DEM directly conjugates to GSH, while BSO inhibits *γ*-glutamylcysteine synthetase, a key enzyme in GSH synthesis. As would be expected from these mechanisms of action, DEM caused a decrease in intracellular cysteine, while BSO increased cysteine levels. The results suggest that negative feedback by intracellular cysteine is an important regulator of system x_c_^−^ in this culture system.

## 1. Introduction

Under normal physiological conditions, the cystine/glutamate exchanger (system x_c_^−^) mediates the transport of cystine into the cell in exchange for releasing glutamate into the extrasynaptic space. The exchange of extracellular cystine and intracellular glutamate occurs in a one-to-one ratio. The function of system x_c_^−^ makes it likely to play an important role in regulating neuronal survival and death. By releasing glutamate, system x_c_^−^ can increase extracellular glutamate levels and potentially cause excitotoxicity. Release of glutamate via system x_c_^−^, from both microglia and astrocytes, has been shown to enhance excitotoxicity of cortical neurons [1–4]. However, through providing cystine to the cell, it regulates the levels of intracellular glutathione (GSH), the main endogenous intracellular antioxidant, and in this way may protect cells against oxidative stress [5, 6].

Not only does system x_c_^−^ act to prevent oxidative stress, but it also appears that such stress is an important trigger for its upregulation. Direct induction of oxidative stress has been shown to upregulate system x_c_^−^ function in a retinal ganglion cell line [7] and in retinal Muller glial cells [8]. Compounds that deplete cellular GSH levels upregulate system x_c_^−^ function in a glioma cell line [9] and in primary astrocytes [10], although there is not always a correlation between depletion of GSH and upregulation of system x_c_^−^ [11].

The first step in the production of GSH in the brain is believed to involve uptake of cystine, primarily into astrocytes [12]. Most of the cystine transported into cortical astrocytes appears to be through system x_c_^−^ [13]. Once in the astrocytes cystine is immediately broken down by thioredoxin reductase 1 into two cysteine molecules [14]. GSH is synthesized via a two-step reaction [15, 16]. First, glutamate and cysteine are catalyzed to *γ*-glutamylcysteine by *γ*-glutamylcysteine synthetase. Then glutathione synthetase combines glycine with *γ*-glutamylcysteine forming GSH. Both glutamate and glycine are highly available in the cells, so the rate-limiting factor in the production of GSH is the levels of cysteine present in the cell [17].

Glutathione can be utilized by cells to reduce reactive oxygen species; for example, superoxide produced as a byproduct of mitochondrial energy production rapidly reacts to form hydrogen peroxide which is then reduced by GSH to form glutathione disulfide (GSSG) and water in a reaction catalyzed by glutathione peroxidase. Glutathione may also be utilized as a xenobiotic detoxicant as has been well characterized involving chemotherapeutics in cancer treatment [18]. That is, GSH can be directly conjugated to exogenous substrates via a disulfide bond with the free sulfhydryl groups; these reactions are directed by a class of enzymes known as glutathione-S-transferases (GSTs) [17, 19]. GSH, GSSG, and the glutathione conjugates are then exported from the cell in a glutathione-dependent manner via multidrug resistance proteins (MRP), specifically MRP1 in the CNS [20, 21]. GSH molecules produced by astrocytes can then be broken down in the extracellular space by glutathione reductase, aminopeptidase N, or *γ*-glutamyl transpeptidase. This metabolism produces the substrate cysteine, which can be taken up and utilized by neurons to produce their own GSH [22–24]. In this way, neurons are dependent on astrocytes to supply the substrate for their GSH production [25]. The importance of cysteine uptake into neurons is indicated by the finding that knocking out the excitatory amino acid transporter-3 (EAAT3) greatly reduces neuronal cysteine uptake and intracellular GSH levels, resulting in decreased viability of hippocampal neurons against hydrogen peroxide insults [26–28].

The current studies used mixed cultures of neurons and astrocytes to be able to incorporate the important interaction between these cell types. The studies involve assessing the effects of two different approaches to deplete cellular GSH. Diethyl maleate (DEM) directly conjugates to GSH while buthionine sulfoximine (BSO) inhibits *γ*-glutamylcysteine synthetase preventing the production of GSH. The studies were designed to determine the effects of these different mechanisms of GSH depletion on system x_c_^−^ function. 

## 2. Materials and Methods

### 2.1. Materials

Timed pregnant Swiss Webster mice were obtained from Charles River Laboratories (Wilmington, DE, USA). Serum was from Atlanta Biologicals (Atlanta, GA, USA). NADPH was from Applichem (Darmstadt, Germany). Radiolabeled ^14^C-cystine was purchased from PerkinElmer (Boston, MA, USA). DCF was from Molecular Probes (Eugene, OR, USA). All other chemicals were from Sigma-Aldrich (St. Louis, MO, USA).

### 2.2. Cortical Cell Cultures

Mixed cortical cell cultures containing glial and neuronal cells were prepared from fetal (15-16 day gestation) mice as previously described [29]. Dissociated cortical cells were plated on 24-well plates (2.0 cm^2^ surface area per well) coated with poly-D-lysine and laminin in Eagles' minimal essential medium (MEM, Earle's salts, supplied glutamine-free) supplemented with 5% (v/v) heat-inactivated horse serum, 5% (v/v) fetal bovine serum, 2 mM glutamine, and D-glucose (total 21 mM). Cultures were maintained in humidified 5% CO_2_ incubators at 37°C with experiments performed on cultures DIV 13–15. Mice were handled in accordance with a protocol approved by our institutional animal care committee and in compliance with the Public Health Service Policy on Humane Care and Use of Laboratory Animals. All efforts were made to minimize animal suffering and reduce the number of animals used. Experiments were performed in media lacking serum (MS), but otherwise identical to the growth media.

### 2.3. Assay of Neuronal Death

Cell death was assessed in cultures by the measurement of lactate dehydrogenase (LDH), released from damaged or destroyed cells, in the extracellular fluid 24 hours after the beginning of the insult. Control LDH levels were subtracted from insult LDH values, and results normalized to 100% neuronal death caused by 500 *μ*M NMDA. Control experiments have shown previously that the efflux of LDH occurring from either necrotic or apoptotic cells is proportional to the number of cells damaged or destroyed [29, 30]. Cultures were also examined visually following trypan blue staining.

### 2.4. 2′,7′-Dichlorofluorescein (DCF) Assay of Oxidative Stress

Oxidative stress was assayed by measuring DCF oxidation using a fluorescent plate reader following a modification of a previous method [31, 32]. Cultures were exposed to 100 *μ*M DEM or BSO for the indicated period of time after which they were exposed to 5-(and-6)-carboxy-2′,7′-dichlorodihydrofluorescein diacetate (carboxy-H_2_DCFDA) (10 *μ*M). The carboxy-H_2_DCFDA is de-esterfied within cells to form a free acid that can then be oxidized to the fluorescent 2′,7′-dichlorofluorescein (DCF). After a 30-minute exposure to carboxy-H_2_DCFDA, cultures were washed 3 times with culture media lacking serum. Fluorescence was then measured using a Fluoroskan Ascent fluorescence plate reader (Thermo Labsystems). The excitation filter was set at 485 nm and emission filter at 538 nm. Background fluorescence (no carboxy-H_2_DCFDA added) was subtracted and the results normalized to control conditions (carboxy-H_2_DCFDA added but no DEM or BSO).

### 2.5. Monochlorobimane (MCB) Assay of Cellular Reduced GSH

Cellular GSH levels were measured by MCB fluorescence. MCB forms a fluorescent compound when it reacts with GSH through a reaction catalyzed by glutathione-S-transferase [33]. Cultures were exposed to the indicated concentrations of DEM or BSO for the indicated period of time after which they were exposed to MCB (10 *μ*M). After 30 minutes, the cultures were excited at a wavelength of 355 nm and emission was measured at a wavelength of 460 nm using a Thermo Labsystems Fluoroskan microplate reader. Background (no MCB added) was subtracted and the results normalized to control (MCB added but no DEM or BSO).

### 2.6. HPLC Analysis of Cellular Cysteine Levels

To assess cysteine concentrations, cultures were exposed to MS containing the indicated drug for 6 or 24 hours. After the indicated time, cultures were washed with balanced salt solution (BSS) and then scraped into 250 *µ*L HPLC mobile phase. Cells were collected into microcentrifuge tubes, sonicated using a probe sonicator and analyzed for protein content using the common BCA method. Once the protein content was determined, the homogenized samples were spun through a centrifugal filter and the resulting protein-free sample was injected onto a Shimadzu HPLC system coupled with an electrochemical detector. Separation was obtained with a reverse phase C-18 column and an ion-pairing mobile phase (50 mM citric acid, 10 mM octane sulfonic acid, pH 2.80, and 1% acetonitrile). Resulting cysteine concentrations were normalized by the protein content and values are reported as percent control.

### 2.7. ^14^C-Cystine Uptake

Radiolabeled cystine uptake was performed as previously described with modifications [34]. Cultures were exposed to MS containing the indicated drug treatments for 40 min, 6 hrs, or 24 hrs. After the drug exposure, cultures were washed into HEPES buffered saline solution and immediately exposed to ^14^C-cystine (0.025 *μ*Ci/mL, 200 nM total cystine) for 20 minutes. Following ^14^C-cystine exposure, cultures were washed with ice cold HEPES buffered saline solution and dissolved in 250 *μ*L warm sodium dodecyl sulfate (0.1%). An aliquot (200 *μ*L) was removed and added to scintillation fluid for counting. Values were normalized to control.

### 2.8. Statistical Analysis

Differences between test groups were examined for statistical significance by means of one-way ANOVA followed by the Bonferroni post hoc analysis, with *P* < 0.05 being considered significant.

## 3. Results

**Figure 1:**
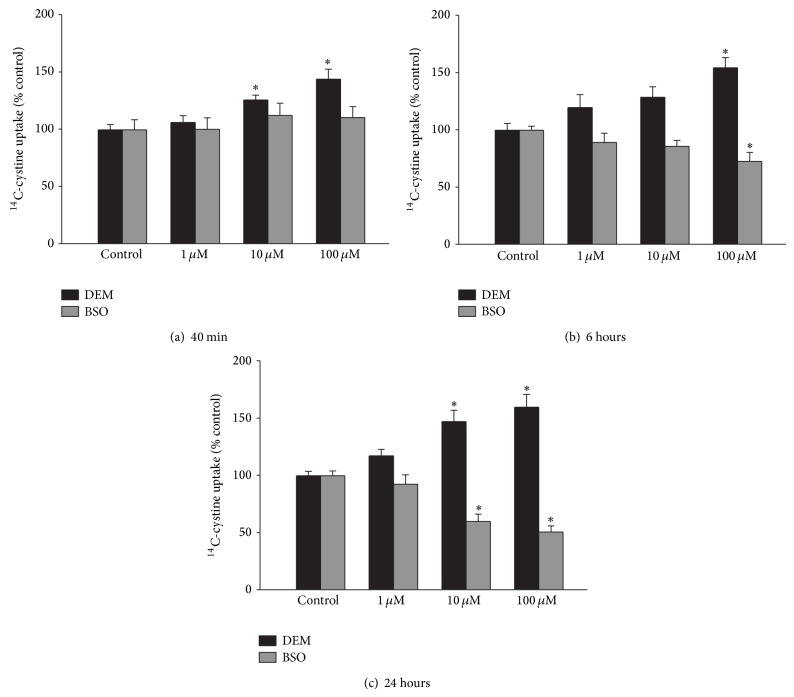
Diethyl maleate (DEM) exposure causes an increase, while buthionine sulfoximine (BSO) exposure causes a decrease, in ^14^C-cystine uptake in mixed cortical cultures. Cultures were exposed to varying concentrations of DEM or BSO for (a) 40 min, (b) 6 hrs, or (c) 24 hrs, after thorough washing, ^14^C-cystine uptake was measured for 20 minutes. Results are expressed as mean + SEM (*n* = 8–16) after normalizing to untreated control uptake. ∗ indicates significant difference from control; *P* < 0.05.

We set out to determine whether depleting cellular GSH alters system x_c_^−^ activity as assessed by measuring ^14^C-cystine uptake in mixed cortical cell cultures. We have shown previously that the large majority of ^14^C-cystine uptake in mixed cortical cultures is mediated by system x_c_^−^ uptake into astrocytes [13]. In the current studies, cellular GSH levels were depleted using two compounds with different mechanisms of action. DEM directly conjugates to GSH, while BSO inhibits GSH synthesis. Varying concentrations of DEM were added to mixed cortical cultures for 40 min, 6 hr, or 24 hrs, with ^14^C-cystine uptake measured for 20 minutes following the exposure. DEM caused a significant increase in ^14^C-cystine uptake at all time points at a concentration of 100 *μ*M, and at 40 min and 24 hrs at the 10 *μ*M concentration (Figure 1). In contrast to DEM, when cultures were exposed to BSO at the same concentrations, BSO did not cause an increase in ^14^C-cystine uptake at any concentration or time point. In fact, BSO at a concentration of 100 *μ*M caused a significant decrease in ^14^C-cystine uptake after 6 hr treatment, while both 10 and 100 *μ*M BSO caused a decrease at 24 hrs (Figure 1).

**Figure 2:**
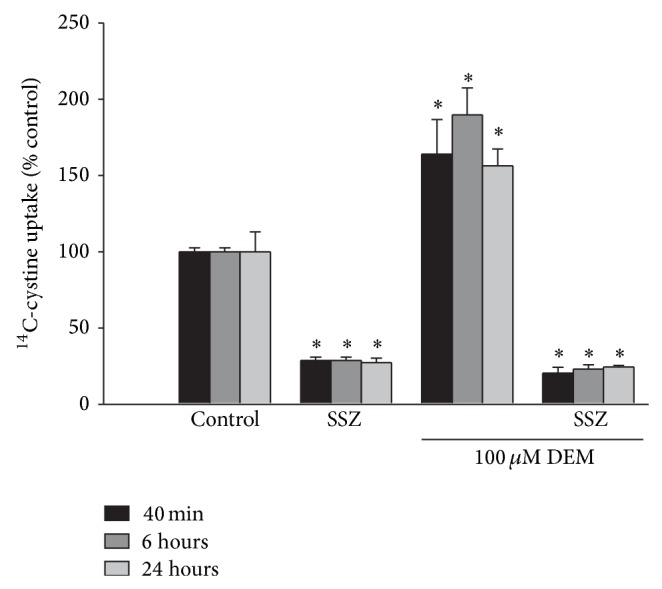
DEM induced increase in ^14^C-cystine uptake is mediated by system x_c_^−^. Cultures were exposed to 100 *μ*M DEM for 40 min, 6 hrs, or 24 hrs, after thorough washing, ^14^C-cystine uptake was measured for 20 minutes with or without the system x_c_^−^ inhibitor sulfasalazine (SSZ) present. Results are expressed as mean + SEM (*n* = 8–16) after normalizing to untreated control uptake. ∗ indicates significant difference from control; *P* < 0.05.

To test whether the increased uptake induced by DEM treatment was mediated by system x_c_^−^, the inhibitor of that system, sulfasalazine (SSZ), was added during the uptake period following exposure to 100 *μ*M DEM. The SSZ treatment completely blocked the increased ^14^C-cystine uptake induced by DEM (Figure 2).

**Figure 3:**
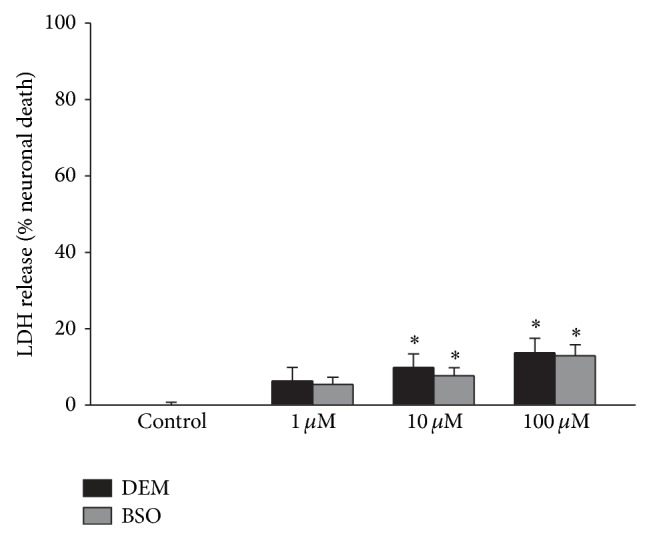
DEM and BSO cause similar low levels of neurotoxicity. Concentration response curve for 24 hr exposure to DEM and BSO on LDH release in primary cortical cultures. Results are expressed as mean + SEM (*n* = 8–16). ∗ indicates significant difference from untreated control; *P* < 0.05.

A potential cause for altered ^14^C-cystine uptake could be toxicity of DEM or BSO. DEM and BSO both caused a small, but significant, level of neurotoxicity after 24 hours at the 100 *μ*M concentrations (Figure 3). Trypan blue staining indicated that the death was selective for neurons (data not shown).

**Figure 4:**
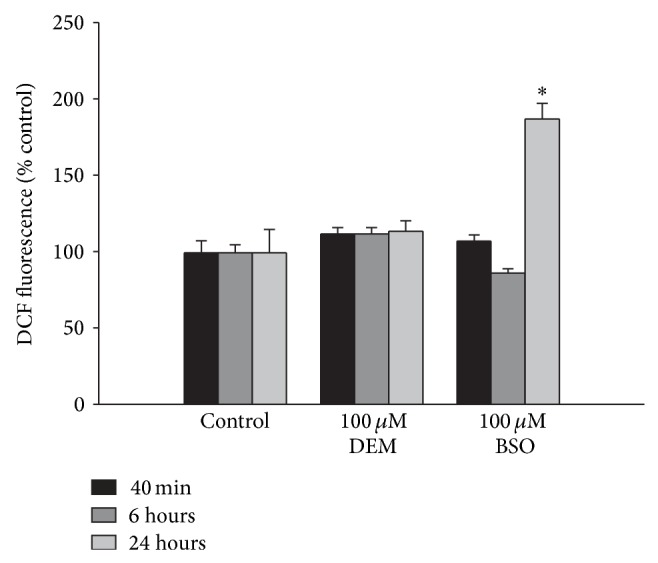
BSO, but not DEM, causes an increase in cellular oxidative stress as measured by DCF fluorescence after 24 hour treatment. Bars show % DCF fluorescence normalized to control fluorescence (mean + SEM, *n* = 8–16). ∗ indicates significant difference from control. *P* < 0.05.

A potential mechanism by which DEM may have caused increased system x_c_^−^ activity is through inducing oxidative stress which has been shown to upregulate system x_c_^−^ [7, 8]. We measured cellular oxidative stress with the compound DCF, which becomes fluorescent when oxidized. Somewhat surprisingly we did not see enhanced DCF fluorescence following 100 *μ*M DEM treatment, while 100 *μ*M BSO only caused an increase following 24 hr treatment (Figure 4).

**Figure 5:**
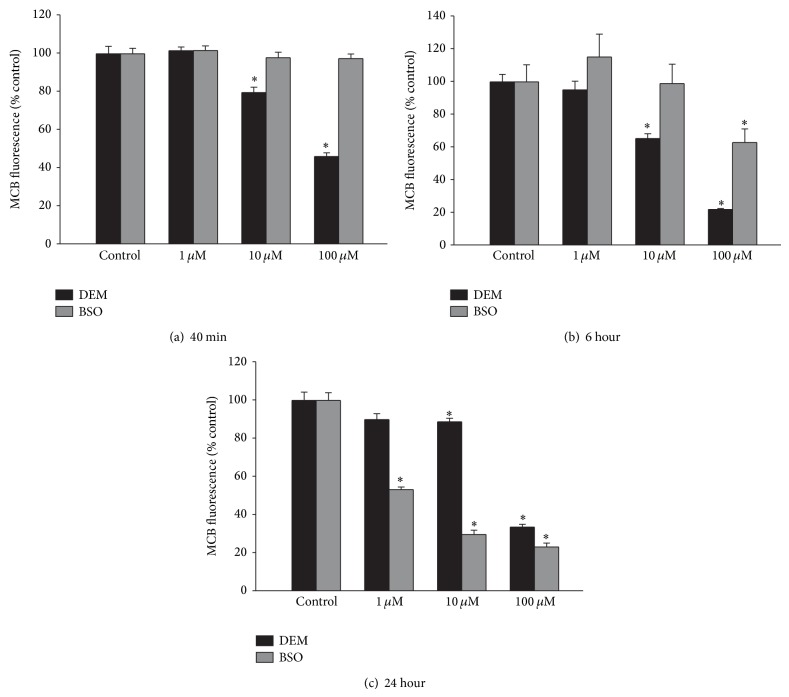
DEM and BSO cause a concentration dependent decrease in cellular glutathione levels. Cultures were exposed to varying concentrations of DEM or BSO for (a) 40 min, (b) 6 hrs, or (c) 24 hrs, after which cellular reduced glutathione was determined by MCB fluorescence. Bars show % MCB fluorescence normalized to control fluorescence (mean + SEM, *n* = 8–16). ∗ indicates significant difference from control. *P* < 0.05.

Another potential mechanism by which DEM may be causing increased system x_c_^−^ function is through causing decreased GSH levels. DEM and BSO treatment both caused a decrease in cellular GSH levels (Figure 5). There were some differences in the decrease, and DEM caused a more rapid decrease in GSH, with a significant decrease at the 40-minute time point, while BSO did not cause a significant decrease until the 6 hr time point. The GSH levels with DEM treatment actually increased from the 6 hr time point to the 24 hr time point, so that, at 24 hrs, BSO caused a greater decrease in GSH levels compared to DEM.

**Figure 6:**
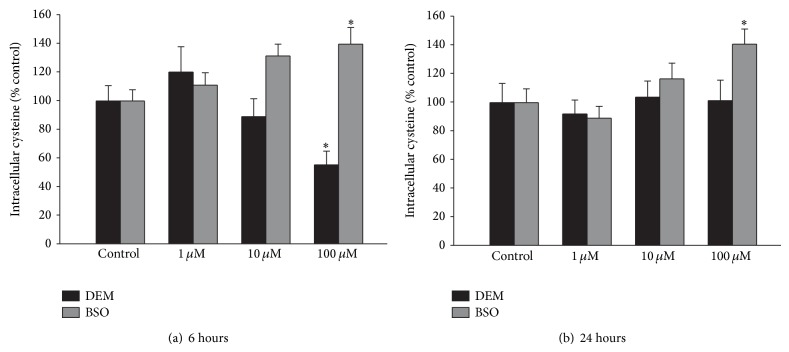
DEM causes an early decrease in cellular cysteine levels, while BSO causes an early and late increase in cellular cysteine levels. Cultures were exposed to varying concentrations of DEM or BSO for (a) 6 hrs or (b) 24 hrs, after which cellular cysteine levels were determined by HPLC. Bars show % cellular cysteine normalized to control (mean + SEM, *n* = 8). ∗ indicates significant difference from control *P* < 0.05.

While DEM and BSO both act to decrease GSH levels, they do so by different mechanisms suggesting the possibility that they may alter cellular cysteine levels differently. We found that 100 *μ*M DEM caused a significant decrease in cellular cysteine levels after 6 hr treatment with the effect disappearing at 24 hours, while 100 *μ*M BSO caused a significant increase in cellular cysteine after 6 and 24 hr treatment (Figure 6).

## 4. Discussion

The regulation of system x_c_^−^ function is proving to be complicated, likely because of its varied functions. Upregulation of system x_c_^−^ by oxidative stress has been well established and this regulation is mechanistically understandable considering that system x_c_^−^ is important for cystine uptake and therefore GSH production. However, system x_c_^−^ function has also been shown to be upregulated by a diverse array of compounds including IL-1*β* [2], erythropoietin [35], FGF-2 [36], IGF-1 [33, 37], TGF-*β* [33], and PACAP [38]. While system x_c_^−^ function has been shown to be decreased by dexamethasone [39], regulation by these diverse compounds may reflect the importance of system x_c_^−^ in regulating not only oxidative stress but also extracellular glutamate. For example, it has been shown that cocaine addiction is associated with impaired system x_c_^−^ function leading to decreased activation of presynaptic group II mGluRs leading to increased synaptic release of glutamate [40, 41]. It is likely that the regulation of system x_c_^−^ by these compounds reflects the importance of regulating both intracellular GSH and extracellular glutamate. Another factor to consider is that there is an interaction between system x_c_^−^ and excitatory amino acid transporters (EAATs) [42]. The possibility that altered EAAT function could change glutamate concentrations and in this way change system x_c_^−^ function exists.

The goal of the current study was to examine in more detail the regulation of system x_c_^−^ by agents that deplete GSH. The previous thinking about such agents is that they upregulated system x_c_^−^ by depletion of cellular GSH, and either this depletion directly stimulated system x_c_^−^ or the resulting increased oxidative stress caused the upregulation. Our results indicate another potential mechanism of regulation. In our studies, simple depletion of cellular GSH did not appear to be the trigger for upregulation of system x_c_^−^. While there were some differences in the time course and concentration dependence of the effects of DEM and BSO treatment on GSH levels, they both caused depletion of GSH and yet they had opposite effects on system x_c_^−^ function. These results are difficult to explain by the different time courses in effects on GSH levels. If BSO caused no effect on system x_c_^−^ function this could potentially be explained by its slower depletion of GSH, but the fact that it actually caused a decrease in system x_c_^−^ activity seems unlikely to be explained by the slower loss of GSH with BSO treatment compared to with DEM treatment. Interestingly, the GSH levels following DEM treatment actually increased from the 6 hr time point to the 24 hr time point (Figure 5). This increase is likely due to the upregulation of system x_c_^−^ under these conditions leading to increased cystine uptake and additional substrate for GSH production.

Toxicity due to treatment with DEM or BSO could be a potential confound when assessing cystine uptake. However, both compounds caused only a small degree of neurotoxicity (10–15%) after 24 hours. This toxicity seems unlikely to play a major role in the results for three reasons. First, DEM and BSO caused similar toxicity but had opposite effects on cystine uptake. Second, the levels of cell death were small compared to the magnitude of changes in cystine uptake. Third, the cell death was selective for neurons, while most of the cystine uptake in this culture system is into astrocytes [13].

The result that DEM did not induce oxidative stress, as measured by DCF fluorescence, suggests that increased oxidative stress is not the mechanism by which DEM upregulates system x_c_^−^ function, particularly since BSO did induce oxidative stress while it actually decreased system x_c_^−^ function. However, it was somewhat surprising that DEM did not induce oxidative stress since it did decrease the levels of GSH. There are a number of possible explanations for this result. First, GSH is not the only antioxidant present in the brain. Under the conditions we were studying it is possible that decreased GSH levels would not lead to enhanced oxidative stress in the cells. Second, the DCF assay does not detect all forms of free radicals [43] and it may be less effective in detecting mitochondria selective oxidative stress [44]. Therefore, while we cannot absolutely conclude that oxidative stress is not the trigger for upregulation of system x_c_^−^ by DEM, the fact that BSO caused a marked increase in oxidative stress while DEM did not, and yet BSO caused a decrease in system x_c_^−^ function, suggesting that factors other than oxidative stress are more important in regulation of system x_c_^−^ in this system.

Our results suggest that, at least for our cell culture system, cysteine is a more important regulator of system x_c_^−^ function than GSH. DEM caused both a decrease in cysteine and GSH levels, consistent with its action to conjugate GSH leading to constant use of cellular cysteine. Therefore, either the decrease in cysteine or GSH could be responsible for the upregulation of system x_c_^−^. However, BSO also decreased GSH but increased cysteine levels, consistent with its action to inhibit *γ*-glutamylcysteine synthetase leading to GSH depletion but buildup of cysteine, and it caused a downregulation of system x_c_^−^ function. In this case, the most likely explanation for the effect is that the buildup of cysteine provides negative feedback on system x_c_^−^ function. This type of regulation makes sense physiologically. The levels of cysteine in the cell are more directly related to the uptake of cystine than they are to the levels of GSH. Glutathione levels in cells could be altered by changes in the function of *γ*-glutamylcysteine synthetase or glutathione synthetase or potentially the availability of glycine or glutamate. Therefore, if system x_c_^−^ was upregulated by GSH depletion, it could be in response to conditions unrelated to the availability of cystine. Additionally, cysteine levels in the brain are 100 times lower than GSH levels [45] and, therefore, rapid changes in their levels are more likely to occur than changes in GSH levels.

Our results are in contrast to those of Seib [10], who found a large increase in system x_c_^−^ function in astrocytes after treatment with BSO. There are two major differences in the culture systems used for the studies. First, we used a mixed neuronal and astrocyte culture, while in the Seib study they used a pure astrocyte culture. The possibility exists that the interaction of neurons with the astrocytes alters the how-cells-regulate system x_c_^−^. Second, their cultures received long-term treatment with a cell permeant form of cAMP prior to the BSO treatment. From their studies, it is clear that GSH levels can also be an important regulator of system x_c_^−^, but that the status of the cells likely determines the relative importance of cysteine or GSH in the regulation. We cannot exclude the possibility that GSH plays a role in regulating system x_c_^−^ even in our culture conditions, but it appears that cysteine levels have a greater effect than GSH on system x_c_^−^ function. The implications this finding has for the role of system x_c_^−^ in disease conditions are not certain. Cysteine levels are not as commonly measured as GSH levels. For example, we have found that system x_c_^−^ function is increased at 70 days of age in the G93A-SOD1 mouse model of ALS [46], a time point at which GSH levels are not yet decreased [47], but intracellular cysteine levels are unknown.

## 5. Conclusions

Our studies indicate that, at least under some conditions, intracellular levels of cysteine are a more important regulator of system x_c_^−^ than intracellular levels of GSH. We did not determine the mechanism of regulation by cysteine, but the redox sensitive transcription factor Nrf2 has been shown to be the main regulator of system x_c_^−^ [11, 48]. This finding puts system x_c_^−^ in the context of it being one factor in the role of Nrf2 as the master regulator of the cellular response to oxidative stress [49]. In conclusion, studies involving assessment of levels of cysteine, GSH, and system x_c_^−^ function during disease conditions will be required to determine the most important regulator of system x_c_^−^ function in disease states.

## Abbreviations

LDH: Lactate dehydrogenase
SSZ: Sulfasalazine
GSH: Glutathione
BSO: Buthionine sulfoximine
DEM: Diethyl maleate
DCF: Dichlorofluorescein
MCB: Monochlorobimane.
